# Breath Analysis: Identification of Potential Volatile Biomarkers for Non-Invasive Diagnosis of Chronic Kidney Disease (CKD)

**DOI:** 10.3390/molecules29194686

**Published:** 2024-10-02

**Authors:** Alessia Di Gilio, Jolanda Palmisani, Marirosa Nisi, Valentina Pizzillo, Marco Fiorentino, Stefania Rotella, Nicola Mastrofilippo, Loreto Gesualdo, Gianluigi de Gennaro

**Affiliations:** 1 Department of Bioscience, Biotechnologies and Environment, University of Bari, 70126 Bari, Italy; 2 Apulian Breath Analysis Center (CeRBA), IRCCS Giovanni Paolo II, 70124 Bari, Italy; 3 Nephrology, Dialysis and Transplantation Unit, Department of Precision and Regenerative Medicine and Ionian Area (DiMePRE-J), University of Bari Aldo Moro, 70121 Bari, Italy; 4 Gastroenterology Unit IRCCS “Saverio de Bellis”, 70013 Castellana Grotte, Italy

**Keywords:** breath analysis, VOCs, diagnosis, chronic kidney disease, renal failure, hemodialysis

## Abstract

Recently, volatile organic compound (VOC) determination in exhaled breath has seen growing interest due to its promising potential in early diagnosis of several pathological conditions, including chronic kidney disease (CKD). Therefore, this study aimed to identify the breath VOC pattern providing an accurate, reproducible and fast CKD diagnosis at early stages of disease. A cross-sectional observational study was carried out, enrolling a total of 30 subjects matched for age and gender. More specifically, the breath samples were collected from (a) 10 patients with end-stage kidney disease (ESKD) before undergoing hemodialysis treatment (DIAL); (b) 10 patients with mild-moderate CKD (G) including 3 patients in stage G2 with mild albuminuria, and 7 patients in stage G3 and (c) 10 healthy controls (CTRL). For each volunteer, an end-tidal exhaled breath sample and an ambient air sample (AA) were collected at the same time on two sorbent tubes by an automated sampling system and analyzed by Thermal Desorption–Gas Chromatography–Mass Spectrometry. A total of 110 VOCs were detected in breath samples but only 42 showed significatively different levels with respect to AA. Nonparametric tests, such as Wilcoxon/Kruskal–Wallis tests, allowed us to identify the most weighting variables able to discriminate between AA, DIAL, G and CTRL breath samples. A promising multivariate data mining approach incorporating only selected variables (showing *p*-values lower than 0.05), such as nonanal, pentane, acetophenone, pentanone, undecane, butanedione, ethyl hexanol and benzene, was developed and cross-validated, providing a prediction accuracy equal to 87% and 100% in identifying patients with both mild–moderate CKD (G) and ESKD (DIAL), respectively.

## 1. Introduction

Impaired kidney function is one of the major public health problems worldwide. In fact, about 13% of the adult population is globally affected by chronic kidney disease (CKD), which is a persistent and progressive impairment of kidney function [1]. CKD is a subtle disease, mainly asymptomatic in early stages and often not detected until the late stage. Moreover, CKD worsens over time, leading to end-stage kidney disease (ESKD) requiring dialysis or kidney transplantation as the only possible strategies to significantly improve the patient’s quality of life and life expectancy. The Kidney Disease: Improving Global Outcomes (KDIGO) group classified kidney disease severity into five stages based on the glomerular filtration rate (GFR): G1 (normal or increased function), G2 (mild GFR decrease), G3 (moderate kidney failure), G4 (severe kidney failure) and finally G5 (ESKD) [2]. Currently, progressively invasive approaches, starting from blood and urine analysis to glomerular filtration rate evaluation up to percutaneous renal biopsy, are used for CKD diagnosis. In many cases, considering the long latency of CKD, a late diagnosis often occurs, thereby increasing the risk of developing ESKD and experiencing poor long-term outcomes. Therefore, a non-invasive, rapid and low-cost method based on the identification of a pattern of disease-related biomarkers could be a useful tool for early diagnosis and follow up of CKD. 

Some studies report an over-production of reactive oxygen species (ROS) and a reduction in antioxidant defenses in CKD [3,4]. In addition, as is well known, kidney disease results in progressive accumulation of water-soluble end-products of cell metabolism which could travel through the bloodstream to reach the pulmonary alveoli and be exhaled in the breath by the alveolar gas exchange mechanism [5,6,7,8,9]. Therefore, it is easy to assume that reduced and impaired kidney function and the increased concentration of ROS in the body may result in a different pattern of volatile organic compounds (VOCs) in exhaled air of CKD patients with respect to healthy subjects. In fact, specific inorganic compounds such as ammonia and several VOCs in human breath were found to be linked to kidney dysfunction [5,10,11,12,13,14]. More specifically, previous published studies reported a relationship between upper breath ammonia and renal failure due to an ineffective removal of waste products in the blood and the accumulation of urea and creatinine that are metabolized and converted into ammonia [10,11,12,13,14]. In the same way, increased levels of dimethylamine (DMA) and trimethylamine (TMA), produced by metabolic conversion of dietary precursors (e.g., choline and carnitine) by gut microbes, were found in the breath of patients with ESKD [6,15,16]. In addition to volatile nitrogen-containing compounds, increased concentrations of isoprene, uremic toxins (cyclohexanone and 2-propenal) and aldehydes were found in nephropathic patients compared to healthy subjects [5,13,17,18,19,20]. Therefore, the identification of increased VOCs in the breath of patients affected by CKD and, thus, of disease-specific biomarker patterns could be useful to (a) obtain an accurate and fast disease diagnosis; (b) implement prevention programs on asymptomatic but potentially at-risk subjects (individuals suffering from hypertension, obesity and diabetes); and (c) provide a more precise evaluation of the disease staging and progression, in turn supporting therapy and disease management [21].

In this context, breath analysis and, especially, the analysis of VOCs in the breath of CKD patients appears to be a promising strategy, as it is completely non-invasive, easily performed and totally safe. Unlike blood and urine, breath samples might be collected frequently, in short time frames and on demand over time. Moreover, breath analysis is proving to be promising in the diagnosis of several pathological conditions because it also offers insights into the health of organs far from the lungs. In fact, various pathophysiological processes altering metabolic and biochemical pathways induce the release into the bloodstream of a different pattern of VOCs respect to physiological conditions. Due to the fast achievement of equilibrium between alveolar air and pulmonary capillary blood, these VOCs in blood can be detected in the breath, and, thus, provide useful information on the health status of an individual [22,23,24]. In addition, the easy repeatability of the breath analysis allows one to evaluate the composition of breath exhaled from potentially at-risk subjects over time, as well as to compare breath samples collected from patients before and after dialysis [3,25,26,27,28,29].

Breath analysis for kidney disease diagnosis and follow up has shown potential in diagnosing kidney disease; however, it is still in early stages and further studies are needed to validate the effectiveness of breath analysis as a diagnostic tool for CKD [3,5,30,31,32,33]. More specifically, it should be highlighted that the studies published until now have mainly focused on the determination of odorous compounds or specific molecules (such as ammonia or amines) in breath samples. Therefore, this study aimed to extend the characterization of uremic breath including a wider panel of VOCs and compare the surely endogeneous VOCs in exhaled breath of CKD patients with those detected in exhaled breath of healthy subjects, also considering ambient air contamination. Finally, in order to develop a screening method for CKD, this study aimed to validate a multivariable predictive model able to discriminate between subjects with renal failure and healthy subjects. 

## 2. Results 

**Figure 1:**
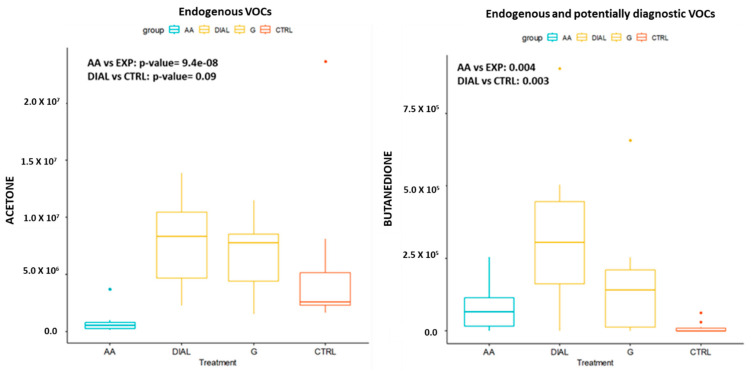
Boxplot and *p*-values obtained by Wilcoxon signed-rank test demonstrating the abundance values related to acetone (e.g., endogenous and non-diagnostic VOC)s and butanedione (e.g., endogenous and potentially diagnostic VOCs).

An overall number of 30 ambient air (AA) samples and 30 breath samples collected from healthy subjects and CKD patients were analyzed. TD-GC/MS chromatograms allowed us to identify about 110 compounds with retention times ranging from 3.6 to 40.1 min and main masses ranging from 39 to 168 m z−1. Among these compounds, 74 VOCs were found in more than 90% of the breath samples. The nonparametric Wilcoxon/Kruskal–Wallis tests (R version 3.6.4) allowed us to identify the most weighting variables to discriminate among DIAL, G and CTRL breath samples, excluding the variables related to ambient air (AA). For example, Figure 1 shows the boxplot and the *p*-value related to acetone, a typical endogenous but non-diagnostic VOC (*p*-value between AA and breath samples equal to 9.8 × 10^−8^; *p*-value between DIAL and CTRL equal to 0.09) and butanedione, an endogenous VOC showing levels significantly different between CRTL and DIAL (*p*-value between AA and breath samples equal to 0.005; *p*-value between DIAL and CTRL equal to 0.004; *p*-value between G and CTRL equal to 0.03). In summary, the abundances of 42 VOCs in breath samples were significantly different with respect to those detected in ambient AAs and, among these, only 15 VOCs showed abundance in breath samples collected from DIAL patients, which is significantly different compared to those detected in CTRL breath samples (Table 1). Moreover, only three VOCs (undecane, butanedione and ethyl hexanol) showed abundance values in breath samples collected from CKD subjects (G) that were significantly different (*p*-value < 0.05) with respect to those detected in CTRL breath samples (Table 1 and Figure 1).

Considering VOCs characterized by *p*-values lower than 0.05 (Table 1), a multivariate statistical approach was applied to collected data. More specifically, a principal component analysis (PCA) was used to provide a partial visualization of data in a reduced-dimension plot. The first two PCs explained 70% of the total variance of the data and, more specifically, PC1 and PC2 accounted for 59% and 11% of the total variance, respectively. According to the loadings of the variables, the most contributing descriptors were benzaldehyde, acetophenone, benzene, nonanal and ethylbenzene for PC1 and pentanone, octane, undecane and ethyl hexanol for PC2.

**Figure 2:**
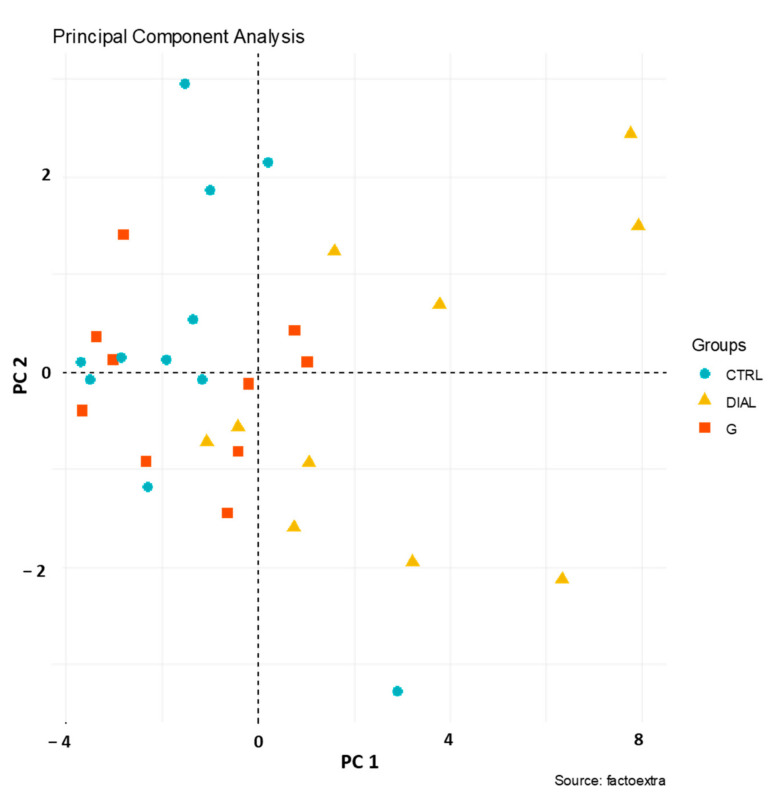
PC1 vs. PC2 scoreplot. Healthy subjects (CTRL) in light blue; dialysis patients (DIAL) in yellow and patients with mild and moderate renal impairment (G) in red.

As shown in Figure 2, poor visual clustering was obtained when the scores of PC1 and PC2 were displayed. More specifically, the PCA allowed us to discriminate between the breath samples collected by hemodialysis patients (DIAL) in yellow and healthy subjects (CTRL) in light blue but did not allow us to effectively distinguish between patients with mild and moderate renal impairment (G2 and G3), shown in red, and healthy subjects, shown in light blue.

**Figure 3:**
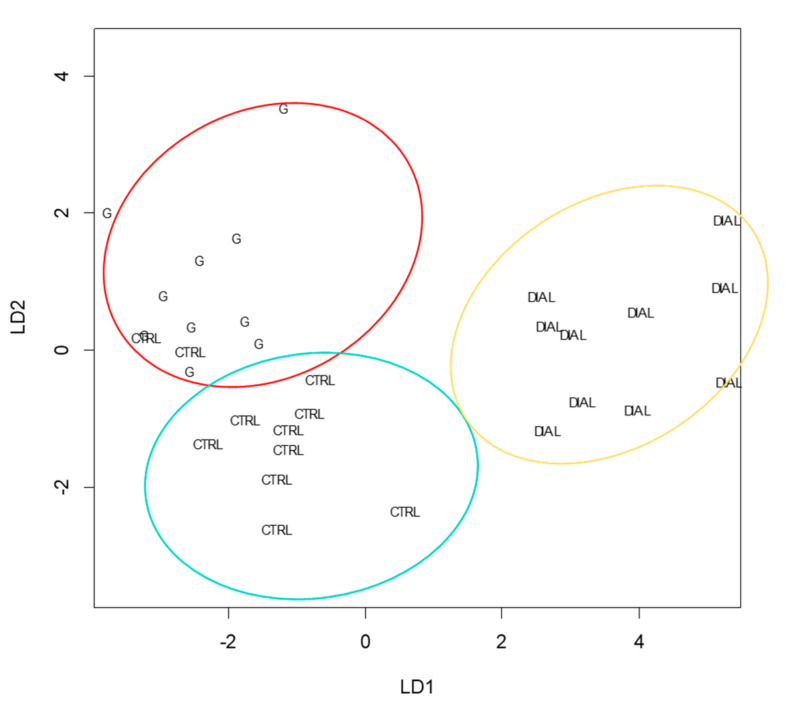
LD1 vs. LD2 scoreplot. Healthy subjects (CTRL) in light blue; dialysis patients (DIAL) in yellow and patients with mild and moderate renal impairment (G) in red.

Therefore, in order to increase and assess the discrimination efficiency among the three different groups, i.e., CTRL, G and DIAL, a multivariate analysis of normalized data was carried out by LDA (R version 3.5.1–MASS package) and two different discriminant functions were computed accounting for 89% and 10% of the total variance in data, respectively. As shown by the scoreplot in Figure 3, LDA is able to discriminate between the three classes of subjects (DIAL, G and CTRL) more effectively than PCA. LD1 is mainly characterized by variables such as nonanal, pentane, acetophenone, pentanone, undecane and butanedione, while LD2 is characterized by ethyl hexanol, ethylbenzene, benzaldehyde and benzene.

In order to validate and optimize the model, the original dataset was randomly divided into two subsets: the training set consisting of 70% of the data and the test set consisting of 30%. Consequently, through an iterative process, the model was trained starting from the training set for which the classification of the sample was known and was subsequently validated by blinded samples (test set). The cross-validation allowed us to evaluate the accuracy of the classification, showing an overall prediction ability equal to 89% with only two CTRLs incorrectly classified as G (false positives) (Figure 3).

**Figure 4:**
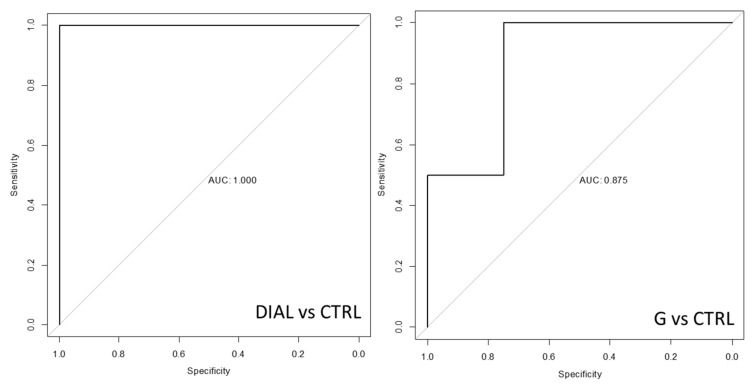
ROC curves.

Using the predicted outcomes, receiver operating characteristic (ROC) curves were constructed (using p-ROC package–R version 3.5.1) for the discrimination both between G and CTRL and between DIAL and CTRL. ROC analysis reported promising results, showing a diagnostic accuracy of 87% and 100% when G vs. CTRL and DIAL vs. CTRL were considered, respectively (Figure 4).

## 3. Discussion

The variables showing significant differences between the three groups (DIAL, CTRL and G) and allowing us to identify patients with both mild and moderate renal functional impairment (G) and hemodialysis patients, on the basis of the developed multivariate data mining approach, are coherent with those reported as biomarkers of CKD in several papers published in the literature to date.

More specifically, according to the results obtained by Michalski et al. 2012 [14] and Si-Hyun Seong et al. 2023 [33], in this study the levels of ketones such as acetophenone, pentanone and butanedione in the breath of CKD patients were found to be significantly higher than the levels detected in AA and in healthy controls. Human urine represents the principal “accumulation site” of ketone bodies in human organisms and their levels in this fluid are particularly elevated in CKD patients. Any impairment of kidney function, in fact, significantly affects the excretion of ketones and, thus, increases their levels in urine and, consequently, in the blood and in the exhaled breath [34,35]. Among the ketones, acetophenone could be linked to the production of phenyl ketones determined by a phenylalanine hydroxylase enzyme (PAH) deficiency due to oxidative stress [9,36]. Several studies, in fact, have linked CKD to an over-production of ROS and a decreased antioxidant defense. For example, Goerl et al. 2013 [4], reported that before hemodialysis, the equilibrium between peroxidative activity determined by free radicals and antioxidative mechanisms appears to be impaired, resulting in oxidative stress [20,31].

Moreover, butanedione is an intermediate metabolite of the acetoin metabolism and, more specifically, it is formed by the decarboxylation of pyruvic acid derived from the metabolism of dietary sugar and citric acid. The increased levels of butanedione in the breath of CKD patients even at the early stages of disease could be linked to insulin resistance, a common alteration in CKD [37,38,39].

In the few studies to date available in the literature, aldehydes such as benzaldehyde and nonanal were found in the breath of patients with CKD [20,31,40]. Several previous studies focused on breath analysis for early diagnosis of different diseases reported aldehydes as breath biomarkers because they are probably linked to oxidative stress and inflammation. Therefore, this finding could explain their role in CKD [41,42]. Moreover, the exogeneous nature of nonanal and benzaldehyde could be excluded because in our study the concentrations of the above-mentioned VOCs in AA were well below alveolar levels. However, as regards benzaldehyde, it should be highlighted that this VOC (as well as benzyl acetate, benzoic acid and benzyl alcohol) is used as a flavoring agent, additive and preservative in food and, thus, is consumed as part of the diet. Moreover, benzyl acetate is quickly hydrolyzed in the body to produce acetic acid and benzyl alcohol which is consequently transformed by the alcohol dehydrogenases in benzaldehyde. In physiological conditions, all these molecules are quickly metabolized by liver enzymes and excreted by urine within a few hours; hence, it is possible that reduced and impaired kidney function determines an accumulation of benzaldehyde in CKD subjects [9,43].

The same considerations apply for Pentane, which has been identified as a biomarker for oxidative stress in numerous studies because it is generated through peroxidation of ω-6- fatty acids in the cell membrane [44,45]. In fact, pentane as well as the other alkane could be the products of ROS-mediated lipid peroxidation [9,44,46,47]. As alkanes, the increased oxidative stress and upregulated CYP450 could determine an increase in alcohols such as 2-Ethylhexanol [9].

Among the potentially diagnostic compounds reported in Table 1, benzene, Toluene, ethylbenzene and Xylene (BTEX) are considered exogenous VOCs linked to industrial emissions and/or vehicle traffic. In any case, these VOCs showed levels in the breath of CKD patients that were significantly higher than those detected in ambient air samples (AA) and in breath samples of healthy controls. These results are coherent with those reported by other authors [4,14]. It is possible that aromatic hydrocarbons such as BTEX can be retained by adipose and/or poorly vascularized tissues due to their lipophilicity and can be slowly and steadily released in the blood. In fact, once inhaled by a subject, VOCs pass into the bloodstream according to their blood/air partition coefficient and are distributed in the different anatomical compartments depending on their chemical nature and, more specifically, according to their affinity for blood, interstitial fluids, moderately perfused tissues and poorly perfused tissues, such as adipose and connective tissues [9,43]. Therefore, levels of these VOCs in the exhaled breath of hemodialysis patients could be significantly higher than the levels detected in the exhaled breath of healthy controls due to the accumulation of exogeneous VOCs in the blood and tissues and a longer pulmonary wash-out time for hemodialysis patients with respect to healthy subjects [4,14,47,48,49,50]. Therefore, considering the levels of VOCs in ambient air and both exogeneous and endogenous VOCs, this study overcomes the limitation of the study conducted by Si-Hyun Seong et al. 2023 [33] and confirms the hypothesis reported by the authors that a reduced and impaired kidney function determines an accumulation of uremic retention metabolites in the body and, thus, results in increased VOCs in breath.

## 4. Materials and Methods

### 4.1. Experimental Study Design

A cross-sectional observational study was carried out by the research group of the Environmental Sustainability Laboratory of the Department of Biosciences, Biotechnologies and Environment in collaboration with the medical team of the Nephrology, Dialysis and Transplantation Unit at the University Hospital Policlinico, Bari. The study was approved by the Local Ethical Committee (Study number 7522, Prot. n. 2914-12/01/2023) and was conducted in accordance with the Declaration of Helsinki. A total number of 30 volunteers aged between 49 and 81 years were enrolled according to inclusion criteria. Subjects younger than 18 years of age, those unable to deliver a breath sample and those affected by malignancy, hepatic disease, asthma, chronic obstructive pulmonary disease and upper or lower respiratory tract infection were not included in the study. More specifically, 10 healthy controls (CTRL group), 10 ESKD patients on chronic hemodialysis (DIAL group) and 10 patients with mild–moderate CKD (n.3 patients in stage G2 and n. 7 in stage G3) (G group) were enrolled. Demographic and clinical data including potential comorbidities, pharmacological treatments and habits (smoking behavior) were collected and recorded for all recruited volunteers. CKD stages were assessed according to KDIGO criteria. Moreover, before breath sampling, all recruited subjects signed an informed consent at the enrolment and refrained from eating, drinking and smoking for at least 12 h before breath sampling. As shown in Table 2, the three different classes of recruited volunteers were approximately matched for age, BMI, sex and pathological conditions. In fact, healthy controls were recruited among subjects affected by hypertension and hypercholesterolemia, which are the main comorbidities with CKD.

### 4.2. Breath Sampling and Analysis

In this study, end-tidal breath samples were collected and, in order to exclude environmental VOCs potentially affecting breath samples and to discriminate between endogenous and exogenous VOCs (environmental contaminants), an ambient air (AA) sample was collected inside the dedicated ambulatory and at the same time as breath sampling.

The end-tidal fraction of the exhaled breath and the AA samples were collected by means of an automated sampler named ‘Mistral’, a medical device developed by an R&D company named Predict s.r.l. (Bari, Italy) with the scientific support of the Department of Biosciences, Biotechnologies and Environment of the University of Bari. Before the breath sampling, the volunteers remained at rest for at least 10 min, guarantying the achievement of an equilibrium between the lungs and ambient air. During this period a preliminary cleaning of the device was carried out by purging all the sampling lines with 1 l of air. The VOCs in ambient air and in the end-tidal fraction of the exhaled breath were separately collected during two different phases of sampling and then directly transferred onto suitable two-bed sorbent tubes packed with Tenax TA and Carbograph 5 TD (Bio-monitoring steel tube, Markes International Ltd., Bridgend, UK) in order to collect a wide range of VOCs (C3-C20). Potential VOC contamination linked to device components and materials has been exhaustively evaluated in our previous studies [43,51].

The collected samples were subsequently analyzed by means of a thermal desorber (UNITY-xr—Markes International Ltd., UK) coupled with a gas chromatograph (GC 7890, Agilent Technologies Inc., Santa Clara, CA, USA) and a mass spectrometer (MS 5975, Agilent Technologies Inc., Santa Clara, CA, USA) and using a previously optimized analytical methodology [9]. Briefly, VOCs adsorbed onto the cartridges were thermally desorbed at 300 °C for 10 min in splitless mode, refocused at 20 °C onto a cold trap specific for wet samples (U-T4WMT-2S Water Management, Markes International Ltd., UK) and, then, promptly transferred by a flash-heating at 300° in a narrow band at the head of the GC column (60 m × 250 µm × 0.25 µm film thickness) characterized by a 5%diphenyl/95%dimethyl polysiloxane stationary phase (VOCOL^®^-Supelco, Merck KGaA, Darmstadt, Germany). A customized mix standard solution including 44 VOCs at concentration of 10 µg/mL in methanol (Ultra Scientific Cus-5997, ULTRA Scientific Italia s.r.l., Bologna, Italy) was analyzed daily to verify the response factors of the GC/MS system over time. Single target ions were extracted from TIC chromatograms (Extracted Ions Chromatograms, EIC mode) using GC/MS post-run analysis software (F.01.03.2357-Agilent Mass Hunter Qualitative Analysis-Agilent Technologies Ltd., USA) in order to improve the number of VOCs identified in breath samples. VOC identification was based on the comparison of the obtained mass spectrum with those included in the National Institute of Standards and Technology library (2017) considering a matching higher than 80% and on the comparison of retention times and ion ratios between VOCs in breath samples and those in standard solution. A total of 110 chromatographic peaks with an intensity higher than five times the baseline signal were integrated and the corresponding areas were included in the dataset. Semi-quantitative analysis was performed in order to consider a wide range of VOCs and not only VOCs included in the standard solution.

### 4.3. Data Analysis

The differences in VOC levels (in terms of compound abundance) among breath (CTRL, DIAL, G) and ambient air (AA) samples were analyzed via paired t-test. Data exhibiting a normal distribution according to the Shapiro–Wilk test were statistically analyzed by parametric tests; otherwise, Wilcoxon/Kruskal–Wallis tests were applied to the dataset in order to compare independent group observations. Nonparametric tests are useful when the data are not necessarily normally distributed and small-size datasets are handled. All statistical tests were performed using the R software (v. 3.6.4, The R Foundation) and only VOCs showing *p*-values less than 0.05 were considered to be statistically different among the groups at a 95% significance level. This approach allowed us to identify both the endogenous VOCs, i.e., chemical compounds showing levels in breath samples (CTRL/DIAL/G) significantly higher than those determined in the correspondent ambient air samples (AA), and endogenous and potentially diagnostic VOCs, i.e., compounds showing levels in breath samples of CKD and hemodialysis patients that were significantly different with respect to those measured in exhaled breath samples collected from healthy controls (CTRL).

Moreover, in order to capture the maximum variability within the data and the most important information contained in the original large dataset, principal component analysis (PCA) was performed by means of R (v. 3.6.4, The R Foundation) using the prcomp function and factoextra package v1.0.7. On the basis of the variables selected by means of the above-mentioned approaches (*p*-value < 0.05), a linear discriminant analysis (LDA) and a cross-validation approach using the lda() function of the MASS package v7.3-61 were also applied to the dataset. LDA is a linear, supervised pattern recognition method that, supported by the classification information regarding every measurement in the training set, finds a linear combination of the input variables able to minimize the variance within each given class and maximize the variance between two classes. The random training set and test set obtained split the main dataset in a 70:30 proportion and were used to perform the cross-validation tests. The accuracy in the classification of subjects as healthy and CKD was quantified through the receiver operating characteristic (ROC) curve calculating the true positive (sensitivity) and the false positive (which is equal to 1− specificity) values.

## 5. Conclusions and Future Perspectives

The advancement of a non-invasive breath test enabling the early identification of toxin accumulation in CKD patients could result in less severe damage and simpler treatment options for patients while also reducing healthcare costs. In this study, a specific breath pattern of VOCs, including nonanal, pentane, acetophenone, pentanone, undecane, butanedione, ethyl hexanol and benzene, was identified and a promising multivariate data mining approach able to recognize and stratify patients with CKD of varying severity (AUC: 1 for hemodialysis patients; AUC: 0.87 for patients affected by mild and moderate renal impairment) was validated. Although the number of collected data points was limited and the developed method requires further validation on a greater amount of data, the results of this study are very interesting and promising. Moreover, it should be highlighted that in this study, ambient air samples were simultaneously collected with breath samples and, thus, the identification of VOC patterns specific to CKD took into account only compounds showing significant differences between levels in breath and AA samples, overcoming one of the biggest limitations of the studies previously conducted on this topic. However, it is important to underline that in this study, the blood and urine levels of the corresponding breath VOCs were not measured. Therefore, the identification of a potential link between internal and external metabolism was not explored and could be a useful perspective for further research on this issue. Such a study would allow us to confirm the results obtained and develop a proper screening method for CKD, while also allowing us to monitor disease progression and evaluate medical treatment efficiency, thereby tailoring therapy on the basis of breath analysis outcomes.

## Figures and Tables

**Table 1:** *P*-value of potentially diagnostic VOCs in breath samples.

Endogenous VOCs	CTRL vs. G	CTRL vs. DIAL
Pentane	0.21	0.03
Octane	0.32	0.02
Eptene	0.72	0.005
Undecane	0.03 *	0.01
Butanedione	0.03 *	0.004
Pentanone	0.33	0.002
Trioxane	0.86	0.01
2-Ethylhexanol	0.02 *	0.0003
Acetophenone	0.85	0.01
Benzaldehyde	0.97	0.02
Nonanal	0.66	0.01
Benzene	0.62	0.02
Toluene	0.97	0.01
Ethylbenzene	0.79	0.0004
Xylene	0.48	0.0006

* Compounds potentially discriminating between CTRL and G.

**Table 2:** Characteristics of the study population.

Total Samples n: 30		CKD (DIAL+G)		
	CTRL (n: 10)	DIAL (n: 10)	CKD Patients (Stage G3) (n: 7)	CKD Patients Stage G2 (n: 3)
Age	63 (49–81)	67 (54–82)	75 (60–83)	66 (65–68)
M:F	6:4 (60% vs. 40%)	6:4 (60% vs. 40%)	6:1 (86% vs. 14%)	6:4 (67% vs. 33%)
BMI mean	25	25	25	27
diabetes	0	2	2	1
hypertension	4	3	3	2
hypercholesterolemia	3	1	2	0

## Data Availability

Data are contained within the article.
